# Endovascular treatment for acute ischemic stroke in patients with versus without atrial fibrillation: a matched-control study

**DOI:** 10.1186/s12883-021-02386-3

**Published:** 2021-09-29

**Authors:** Xu Tong, Shijing Li, Wei Liu, Zeguang Ren, Raynald Liu, Baixue Jia, Xuelei Zhang, Xiaochuan Huo, Gang Luo, Gaoting Ma, Anxin Wang, Yilong Wang, Yongjun Wang, Zhongrong Miao, Dapeng Mo, on behalf of ANGEL-ACT study group, Zhongrong Miao, Liqiang Gui, Cunfeng Song, Ya Peng, Jin Wu, Shijun Zhao, Junfeng Zhao, Zhiming Zhou, Yongli Li, Ping Jing, Lei Yang, Yajie Liu, Qingshi Zhao, Yan Liu, Xiaoxiang Peng, Qingchun Gao, Zaiyu Guo, Wenhuo Chen, Weirong Li, Xiaojiang Cheng, Yun Xu, Yongqiang Zhang, Guilian Zhang, Yijiu Lu, Xinyu Lu, Dengxiang Wang, Yan Wang, Hao Li, Yang Hua, Deqin Geng, Haicheng Yuan, Hongwei Wang, Haihua Yang, Zengwu Wang, Liping Wei, Xuancong Liufu, Xiangqun Shi, Juntao Li, Wenwu Yang, Wenji Jing, Xiang Yong, Leyuan Wang, Chunlei Li, Yibin Cao, Qingfeng Zhu, Peng Zhang, Xiang Luo, Shengli Chen, Wen Wu Peng, Lixin Wang, Xue Wen, Shugui Shi, Wanming Wang, Wang Bo, Pu Yuan, Dong Wang, Haitao Guan, Wenbao Liang, Daliang Ma, Long Chen, Yan Xiao, Xiangdong Xie, Zhonghua Shi, Xiangjun Zeng, Fanfan Su, Ming Ze Chang, Jijun Yin, Hongxia Sun, Chong Li, Yong Bi, Gang Xie, Yuwu Zhao, Chao Wang, Peng Zhang, Xianjun Wang, Dongqun Li, Hui Liang, Zhonglun Chen, Yan Wang, Yuxin Wang, Lin Yin, Hong Kai Qiu, Jun Wei, Yaxuan Sun, Xiaoya Feng, Weihua Wu, Lianbo Gao, Zhibing Ai, Lan Tan, Li Ding, Qilong Liang, Zhimin Wang, Jianwen Yang, Ping Xu, Wei Dong, Quanle Zheng, Zhenyun Zhu, Liyue Zhao, Qingbo Meng, Yuqing Wei, Xianglin Chen, Wei Wang, Dong Sun, Yongxing Yan, Guangxiong Yuan, Yadong Yang, Jianfeng Zhou, Zhi Yang, Zhenzhong Zhang, Ning Guan, Huihong Wang

**Affiliations:** 1 Department of Interventional Neuroradiology, Beijing Tiantan Hospital, Capital Medical University, No.119 South 4th Ring West Road, Fengtai District, Beijing, China; 2 Department of General Practice, Beijing Mentougou District Hospital, Beijing, China; 3 Center for Medical Device Evaluation, National Medical Product Administration, Beijing, China; 4 Department of Neurosurgery, University of South Florida, Tampa, Florida USA; 5 Beijing Institute of Brain Disorders, Capital Medical University, Beijing, China; 6 China National Clinical Research Center for Neurological Diseases, Beijing Tiantan Hospital, Capital Medical University, Beijing, China; 7 Department of Neurology, Beijing Tiantan Hospital, Capital Medical University, Beijing, China

**Keywords:** Atrial fibrillation, Endovascular treatment, Ischemic stroke, Propensity score matching

## Abstract

**Background and objective:**

The effect of atrial fibrillation (AF) on outcomes of endovascular treatment (EVT) for acute ischemic stroke (AIS) is controversial. This study aimed to investigate the association of AF with outcomes after EVT in AIS patients.

**Methods:**

Subjects were selected from ANGEL-ACT registry (Endovascular Treatment Key Technique and Emergency Work Flow Improvement of Acute Ischemic Stroke) - a prospective consecutive cohort of AIS patients undergoing EVT at 111 hospitals in China between November 2017 and March 2019, and then grouped according to having a history of AF or not. After 1:1 propensity score matching, the outcome measures including the 90-day modified Rankin Scale (mRS) score, successful recanalization after final attempt, symptomatic intracranial hemorrhage (ICH) within 24 h, and death within 90 days were compared.

**Results:**

A total of 1755 patients, 550 with AF and 1205 without AF, were included. Among 407 pairs of patients identified after matching, no significant differences were found in the mRS score (median: 3 vs. 3 points; *P* = 0.29), successful recanalization (87.2 vs. 85.3%; *P* = 0.42), symptomatic ICH (9. 4 vs. 9.1%; *P* = 0.86) and death (16.3 vs. 18.4%; *P* = 0.44) between patients with and without AF.

**Conclusion:**

The findings of this matched-control study show comparable outcomes of EVT in Chinese AIS patients with and without AF, which do not support withholding EVT in patients with both AIS and AF.

**Trial registration:**

NCT03370939

First registration date: 28/09/2017

First posted date: 13/12/2017

## Introduction

Atrial fibrillation (AF), as the most common cause of cardioembolic stroke, is associated with a 4-5 times increased risk of acute ischemic stroke (AIS) and accounts for approximately 30–40% of all acute large vessel occlusion (LVO) [1–8]. Patients with AF-related stroke are older, have greater burden of comorbidities and worse neurological deficits, thus have a higher probability of disability or mortality after usual care [9–12]. Furthermore, intravenous thrombolysis (IVT) is less effective on both recanalization and clinical outcome but also increases the risk of intracranial hemorrhage (ICH) in patients with AF. The poor response to IVT could be partly explained by the pathophysiology of AF-related stroke, such as the gaps between patients with and without AF in terms of embolic size and components, collateral status, infarct core volume, and stroke progression [13, 14].

Endovascular treatment (EVT) represented by mechanical thrombectomy with stent-retriever or aspiration catheter has become the standard treatment for selected patients with AIS due to intracranial proximal LVO [15]. However, limited data and conflicting results exist regarding the role of AF on procedural and clinical outcomes after EVT [16–21]. To address this issue and on the hypothesis that the modification of AF was attributed to the effect of case mix; in other words, AF might not independently affect any outcome in EVT-treated patients after adjusting for possible confounders. We therefore performed a matched-control analysis based on a prospective nationwide registry database to assess whether the technical success and functional outcomes differ in LVO patients with and without AF after receiving EVT.

## Methods

### Study population

Data were extracted from ANGEL-ACT (Endovascular Treatment Key Technique and Emergency Work Flow Improvement of Acute Ischemic Stroke), a prospective nationwide registry of 1793 consecutive patients with AIS caused by LVO undergoing EVT in 111 hospitals in China between November 2017 and March 2019. Full methods of the registry, such as inclusion/exclusion criteria and data collection standards, have been reported earlier [22]. The protocol was approved by the ethics committees of all centers, and all participants (or legal representatives) provided written informed consent. The study procedures were in accordance with the 1964 Helsinki declaration and its later amendments.

In this analysis, patients with missing baseline or procedure data in Table 1 were excluded, and the remainder cases were divided into two groups based on whether they had pre-existing AF, identified by previous medical records.

**Table 1:** Baseline and procedure characteristics of patients with AF versus without AF

Baseline and procedure variables	Pre-matched population (***n*** = 1755)				Post-matched population (***n*** = 814)			
	With AF (***n*** = 550)	Without AF (***n*** = 1205)	SD (%)	***P***-value	With AF (***n*** = 407)	Without AF (***n*** = 407)	SD (%)	***P***-value
Age, median (IQR), years	71 (64–78)	63 (54–70)	72.0	< 0.01	69 (62–76)	68 (61–75)	4.3	0.32
Male sex	246 (44.7)	910 (75.5)	66.2	< 0.01	213 (52.3)	221 (54.3)	3.9	0.57
History of hypertension	333 (60.6)	673 (55.9)	9.5	0.07	232 (57.0)	245 (60.2)	6.5	0.35
History of diabetes mellitus	99 (18.0)	225 (18.7)	1.8	0.74	73 (17.9)	82 (20. 2)	5.6	0.42
Prior ischemic stroke	130 (23.6)	207 (17.2)	16.1	< 0.01	85 (20.9)	88 (21.6)	1.8	0.80
Pre-stroke mRS score ≥ 1	84 (15.3)	146 (12.1)	9.2	0.07	55 (13.5)	60 (14.7)	3.5	0.61
Cigarette smoking			56.5	< 0.01			7.3	0.27
Never Smoker	420 (76.4)	629 (52.2)			291	285		
Ex-smoker	44 (8.0)	89 (7.4)			37	28		
Current smoker	86 (15.6)	487 (40. 4)			79	94		
Systolic blood pressure, median (IQR), mmHg	145 (130–160)	145 (132–162)	7.5	0. 21	145 (130–160)	145 (130–160)	2.1	0.95
NIHSS score, median (IQR)	18 (14–22)	15 (11–21)	29.5	< 0.01	17 (13–21)	17 (13–22)	2.6	0.87
ASPECTS, median (IQR) ^a^	10 (7–10)	9 (7–10)	13.1	< 0.01	10 (7–10)	10 (7–10)	1.5	0.91
Occlusion site			46.4	< 0.01			9.1	0.20
Internal carotid artery	166 (30.2)	279 (23.2)			111 (27.3)	116 (28.5)		
Middle cerebral artery M1 segment	266 (48.4)	493 (40.9)			197 (48.4)	187 (45.9)		
Middle cerebral artery M2 segment	59 (10.7)	91 (7.6)			47 (11.6)	39 (9.6)		
Vertebro-basilar artery	49 (8.9)	313 (26.0)			42 (10.3)	60 (14.7)		
Other intracranial arteries ^b^	10 (1.8)	29 (2.4)			10 (2.5)	5 (1. 2)		
Prior use of antiplatelet agents	101 (18.4)	187 (15.5)	7.8	0.14	75 (18.4)	73 (17.9)	1.3	0.86
Prior use of anticoagulants	51 (9.3)	20 (1.7)	34.0	< 0.01	19 (4. 7)	15 (3.7)	4.9	0.48
Prior intravenous thrombolysis	145 (26.4)	368 (30.5)	9.3	0.07	115 (28.3)	102 (25.1)	7.2	0.30
Type of anesthesia			16.8	0.01			4.1	0.55
Local anesthesia only	265 (48.2)	500 (41.5)			190 (46.7)	184 (45.2)		
Local anesthesia plus sedation	92 (16.7)	190 (15.8)			68 (16.7)	60 (14.7)		
General anesthesia	193 (35.1)	515 (42.7)			149 (36.6)	163 (40.1)		
Stent-retriever thrombectomy	385 (70.0)	834 (69.2)	1.7	0.74	284 (69.8)	289 (71.0)	2.7	0.70
Aspiration thrombectomy	14 (2. 6)	40 (3.3)	4.6	0.38	13 (3.2)	15 (3.7)	2.7	0.70
Stent-retriever plus aspiration thrombectomy	124 (22.6)	180 (14.9)	19.6	< 0.01	83 (20.4)	77 (18.9)	3.7	0.60
Pass number of thrombectomy, median (IQR)	2 (1–3)	1 (1, 2)	40.8	< 0.01	2 (1–3)	2 (1–3)	1.4	0.79
Emergency angioplasty/stenting	45 (8.2)	471 (39.1)	78.1	< 0.01	45 (11.1)	57 (14.0)	8.9	0.20
Intra-arterial thrombolysis	33 (6.0)	111 (9.2)	12.1	0.02	31 (7.6)	31 (7.6)	0.0	1.00
Intra-procedural use of tirofiban	201 (36.6)	712 (59.1)	46.3	< 0.01	167 (41.0)	186 (45.7)	9.3	0.18
Intra-procedural use of heparin	251 (45.6)	606 (50.3)	9.3	0.07	187 (46.0)	178 (43.7)	4.5	0.53
Onset-to-puncture time, median (IQR), min	260 (195–370)	325 (225–484)	30.8	< 0.01	284 (200–390)	290 (210–410)	6.0	0.22
Puncture-to-recanalization time, median (IQR), min	80 (50–120)	89 (54–135)	11.2	0.01	79 (50–120)	87 (53–128)	5.3	0.19

*Abbreviations*: *AF* atrial fibrillation, *ASPECTS* Alberta Stroke Program Early CT Score, *IQR* interquartile range, *mRS* modified Rankin Scale, *NIHSS* National Institutes of Health Stroke Scale, *pc-ASPECTS* posterior circulation Alberta Stroke Program Early CT Score, *SD* standardized difference

Values are numbers with percentages in parentheses, unless indicated otherwise

^a^ASPECTS for anterior circulation stroke, and pc-ASPECTS for posterior circulation stroke

^b^including anterior cerebral artery A1/A2 segments, posterior cerebral artery P1 segment

### Outcome measures

The primary outcome was the 90-day modified Rankin Scale (mRS) score assessed by trained and independent investigators. The secondary outcomes included successful recanalization (modified Thrombolysis in Cerebral Infarction [mTICI] of 2b-3) after first and final attempt, complete recanalization (mTICI of 3) after final attempt, [23] the proportions of mRS 0–1, 0–2 and 0–3 at 90 days. The safety outcomes were intra-procedural complications (e.g., new territorial embolization, arterial perforation, arterial dissection, vasospasm requiring treatment and in-stent thrombosis), any ICH, parenchymal hematoma type 2 (PH2) and symptomatic ICH within 24 hours according to the Heidelberg Bleeding Classification, [24] and death within 90 days.

### Statistical analysis

Data were displayed as median (interquartile range [IQR]) or frequency (percentage). Univariable comparisons of baseline characteristics between patients with and without AF were performed using Mann-Whitney or Pearson’s chi-square tests. To improve the comparability between the two groups, a 1:1 propensity score matching (PSM) was performed by using a caliper distance of 0.05 [25]. For comparing the outcomes between both groups, the odds ratios (OR) or common OR with their 95% confidence intervals (CI) were calculated using a binary or ordinal logistic regression model, if applicable. Significance level was set to α = 0.05 (2-sided). Statistical analyses were conducted with SAS software version 9.4 (SAS Institute Inc., Cary, NC).

## Results

**Fig. 1:**
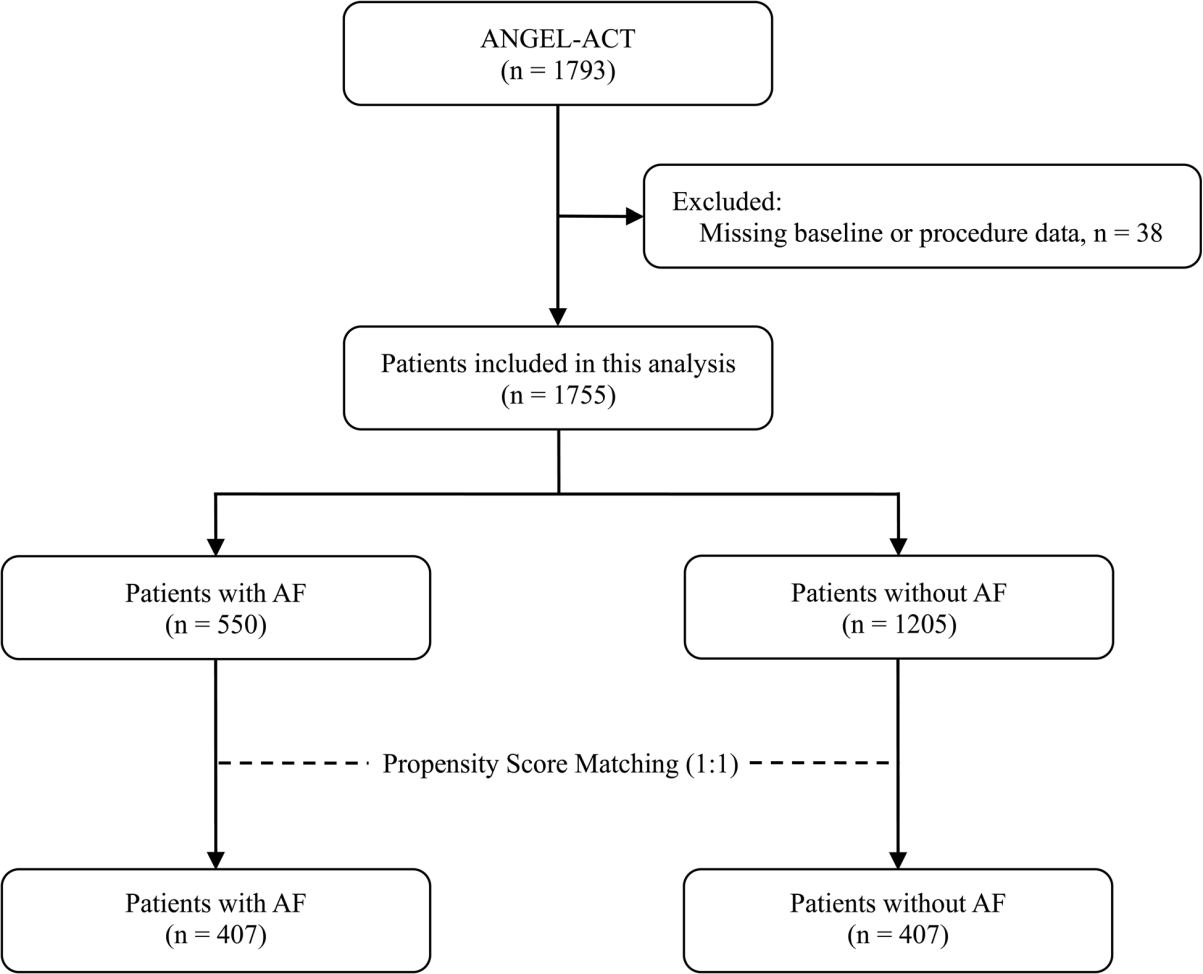
Flow chart of patient selection. Abbreviations: AF = atrial fibrillation, ANGEL-ACT = Endovascular Treatment Key Technique and Emergency Work Flow Improvement of Acute Ischemic Stroke

Among 1793 patients enrolled in the ANGEL-ACT registry, 38 patients were excluded due to missing baseline or procedure information, a total of 1755 patients were included in this analysis, including 550 cases with AF and 1205 without AF. After PSM, 814 patients were identified (Fig. 1**)**.

As shown in Table 1, there were significant differences in many baseline and procedure characteristics between pre-matched patients with and without AF. For example, patients with AF were 8 years older, had 3 points higher NIHSS scores, were more frequently given anticoagulants before stroke onset, and received more passes of thrombectomy than those without AF; while patients with AF had lower proportions of male, current smoker, and vertebro-basilar artery occlusion, were less often given tirofiban during the procedure and emergency angioplasty/stenting, and experienced 65 min shorter onset-to-puncture time than those without AF (all *P*-values < 0.01). After PSM, all baseline and procedure characteristics between groups were well-balanced (Table 1).

Comparisons of outcome measures between patients with and without AF were presented in Table 2. Before matching, there was no significant difference in recanalization rates between the two groups, but patients with AF had a higher 90-day mRS score (*P* < 0.01) and higher risks of intra-procedural complications (*P* = 0.02), hemorrhagic transformations within 24 hours (all *P* < 0.01), and death within 90 days (*P* = 0.01), whereas they had lower proportions of mRS 0–1, 0–2, and 0–3 points at 90 days (all *P* < 0.01). After matching, the difference in the primary outcome - 90-day mRS score no longer existed between patients with and without AF (median: 3 vs. 3 points; *P* = 0.29). In addition, all differences in secondary and safety outcomes that differed between both groups before matching also disappeared.

**Table 2:** Outcome measures of patients with AF versus without AF

Outcome variables	Pre-matched population (***n*** = 1755)				Post-matched population (***n*** = 814)			
	With AF (***n*** = 550)	Without AF (***n*** = 1205)	Univariable analysis		With AF (***n*** = 407)	Without AF (***n*** = 407)	Univariable analysis	
			Effect size (95% CI)	P-value			Effect size (95% CI)	***P***-value
**Primary outcome**								
mRS at 90 d, median (IQR)	4 (1–5)	3 (0–5)	0.59 (0.47–0.74) ^a^	< 0.01	3 (0–5)	3 (1–5)	1. 16 (0.82–1.52) ^a^	0.29
**Secondary outcomes**								
Successful recanalization after first attempt ^c^	267/550 (48.6)	588/1205 (48.8)	0.99 (0.81–1. 21) ^b^	0.92	209/407 (51.4)	190/407 (46.7)	1. 21 (0.92–1.59) ^b^	0.18
Successful recanalization after final attempt ^c^	479/550 (87.1)	1065/1205 (88.4)	0.89 (0.65–1. 20) ^b^	0.44	355/407 (87.2)	347/407 (85.3)	1. 18 (0.79–1.76) ^b^	0.42
Complete recanalization after final attempt ^d^	376/550 (68.4)	789/1205 (65.5)	1. 14 (0.92–1.41) ^b^	0. 24	279/407 (68.6)	264/407 (64.9)	1. 18 (0.88–1.58) ^b^	0.27
mRS 0–1 at 90 d	174/518 (33.6)	521/1162 (44.8)	0.62 (0.50–0.77) ^b^	< 0.01	143/387 (37.0)	143/386 (37.1)	1.00 (0.74–1.33) ^b^	0.98
mRS 0–2 at 90 d	195/518 (37.6)	565/1162 (48.6)	0.64 (0.52–0.79) ^b^	< 0.01	160/387 (41.3)	155/386 (40.2)	1.05 (0.79–1.40) ^b^	0.74
mRS 0–3 at 90 d	252/518 (48.7)	676/1162 (58.2)	0.68 (0.55–0.84) ^b^	< 0.01	208/387 (53.8)	192/386 (49.7)	1. 17 (0.89–1.56) ^b^	0.27
**Safety outcomes**								
Intra-procedural complications ^e^	63/550 (11.5)	95/1205 (7.9)	1.51 (1.08–2. 12) ^b^	0.02	43/407 (10.6)	36/407 (8.8)	1. 22 (0.76–1.94) ^b^	0.41
Any ICH within 24 h	158/516 (30.6)	222/1163 (19.1)	1.87 (1.48–2.37) ^b^	< 0.01	106/384 (27.6)	95/388 (24.5)	1. 18 (0.85–1.62) ^b^	0.32
PH2 within 24 h ^f^	35/516 (6.8)	41/1163 (3.5)	1.99 (1. 25-3. 17) ^b^	< 0.01	25/384 (6.5)	23/388 (5.9)	1. 11 (0.62–1.98) ^b^	0.74
Symptomatic ICH within 24 h ^f^	54/513 (10.5)	70/1156 (6.1)	1.83 (1. 26-2.65) ^b^	< 0.01	36/381 (9.4)	35/386 (9.1)	1.05 (0.64–1.71) ^b^	0.86
Death within 90 d	100/518 (19.3)	162/1162 (13.9)	1.48 (1. 12-1.94) ^b^	0.01	63/387 (16.3)	71/386 (18.4)	0.86 (0.59–1. 25) ^b^	0.44

*Abbreviations*: *AF* atrial fibrillation, *CI* confidence interval, *ICH* intracranial hemorrhage, *IQR* interquartile range, *mRS* modified Rankin Scale, *mTICI* modified Thrombolysis in Cerebral Infarction, *OR* odds ratio, *PH2* parenchymal hematoma type 2

Data are shown as the event number/total number (%), unless otherwise indicated

^a^The common OR values were calculated using an ordinal logistic regression model and indicated the odds of improvement of 1 point on the mRS at 90 days

^b^The OR values were calculated using a binary logistic regression model

^c^Defined as mTICI of 2b-3

^d^Defined as mTICI of 3

^e^Including new territorial embolization, arterial perforation, arterial dissection, vasospasm requiring treatment and in-stent thrombosis

^f^ccording to the Heidelberg Bleeding Classification

## Discussion

This real-world registry study in China found that patients with AF were older, had more severe symptoms on admission, a lower proportion of posterior circulation occlusions, and a shorter time from onset to puncture. After matching for baseline characteristics using propensity scores, AF was not independently associated with 90-day functional outcomes, recanalization rates, and intra-procedural complications.

A subgroup analysis of the MR CLEAN trial (Multicenter Randomized Clinical Trial of Endovascular Treatment for Acute Ischemic Stroke in the Netherlands) showed a trend towards a decreased treatment effect of EVT in patients with AF. However, the sample size of AF patients in their study was rather small, thus no definite conclusion could be drawn [16]. A subsequent meta-analysis from the HERMES collaboration (Highly Effective Reperfusion Evaluated in Multiple Endovascular Stroke Trials) demonstrated no interaction between AF and functional outcomes after EVT, but found a trend towards a lower rate of symptomatic ICH in AIS patients with AF (3.4% in AF patients vs. 4.5% in non-AF patients), which might be related to the lower percentage of pre-treatment with IVT (76.3% in AF patients vs. 90.6% in non-AF patients). This is probably mainly due to the fact that patients with AF are more likely to taking oral anticoagulants, which is a contraindication for the administration of tPA [17]. Conversely, a post-hoc analysis of a multi-center head-to-head clinical trial revealed that AF was an independent risk factor for any ICH in AIS patients undergoing stent-retriever thrombectomy, which was partly attributable to the adjusted anticoagulation status and more retrieval attempts by mediation analyses [18]. Furthermore, a national registry study assessing post-thrombectomy outcomes found no difference in either in-hospital or discharge outcomes between matched patients with or without AF, [19] whereas two other studies suggested faster procedural time, fewer passes, higher rates of first pass effect, successful reperfusion and good functional outcome with AF-related stroke [20, 21].

Previous observations found patients with AIS caused by AF tend to have more bleedings and worse outcomes after EVT than those without AF [16, 18]. However, special cautions should be taken when interpreting these results, such a statement could lead to misconclusions to suspecting or even denying EVT to patients with AF. We may expect that AIS caused by a sudden embolus from the cardiovascular circulation can progress faster than AIS caused by progressive carotid or intracranial artery stenosis, where there may be time for development of collaterals [26]. In this study, patients with AF were treated about 1 hour earlier (median time from onset to puncture: 260 min vs. 325 min) compared to those without AF, suggesting a faster infarct growth rate and a stronger time dependence of reperfusion therapy in AF-related stroke.

Strengths of this study were the large sample size of enrolled patients (*n* = 1755) and the high prevalence of AF (31. 3%), resulting in more reliable estimations. Also, comparison of outcomes after PSM was a strength. Finally, all radiological and clinical outcomes in this analysis were centrally adjudicated by the independent imaging core laboratory or clinical events committee, except those intra-procedural complications were locally scored by site investigators. Nevertheless, our study has some limitations. First, the collateral status has been shown to be an excellent predictor of stroke outcomes, [27] so a major limitation of this study is the lack of assessment of collateral status, which has been postulated as a possible reason for difference in functional outcomes post-EVT of LVO patients with vs. without AF [28, 29]. Second, this study was conducted in Chinese population, where the prevalence of intracranial atherosclerotic disease (ICAD) is very high [30]. In this context, an underlying ICAD stenotic lesion is often cited as a possible reason for immediate re-occlusion after thrombectomy that results in bailout intracranial angioplasty or stenting, thus potentially having an impact on the outcomes [31]. Our findings should be interpreted with caution and could not easily be extrapolated to other populations. Third, patients with AF may have more comorbidities (e.g., decreased ejection fraction, valvular heart disease, other organ failure), larger infarct core, and different texture of thrombus compared to those without AF. However, these variables were not collected in the ANGEL-ACT registry, so their confounding effects could not be ruled out. Finally, no information on antithrombotic therapy from post-procedure to discharge, treatment adherence and rehabilitation training after discharge was recorded, therefore limiting comments on the association between them and functional outcomes.

## Conclusion

The present study found no difference in the radiological and clinical outcomes following EVT between Chinese AIS patients with and without AF, implying AF status should not hamper the decision making to proceed to EVT. Furthermore, our results were in contrast to the increased hemorrhage rates and worse functional outcomes observed in AF-related stroke treated with supportive care or IVT. It is known that thrombolysis is less used in patients with AF-related LVO and, if used, has only limited effect. Thus, the fact is EVT might be the best chance for these patients.

## Data Availability

The data that support the findings of this study are available from the corresponding author Dapeng Mo (bjttmodp@163.com) or Zhongrong Miao (zhongrongm@163.com) upon reasonable request.

## Abbreviations

AF: Atrial fibrillation
AIS: Acute ischemic stroke
ANGEL-ACT: Endovascular Treatment Key Technique and Emergency Work Flow Improvement of Acute Ischemic Stroke
ASPECTS: Alberta Stroke Program Early CT Score
CI: Confidence interval
EVT: Endovascular treatment
HERMES: Highly Effective Reperfusion Evaluated in Multiple Endovascular Stroke Trials
ICH: Intracranial hemorrhage
IQR: Interquartile range
IVT: Intravenous thrombolysis
LVO: Large vessel occlusion
MR CLEAN: Multicenter Randomized Clinical Trial of Endovascular Treatment for Acute Ischemic Stroke in the Netherlands
mRS: Modified Rankin Scale
mTICI: Modified Thrombolysis in Cerebral Infarction
NIHSS: National Institutes of Health Stroke Scale
OR: Odds ratio
pc-ASPECTS: Posterior circulation Alberta Stroke Program Early CT Score
PH2: Parenchymal hematoma type 2
PSM: Propensity score matching
SD: Standardized difference

## References

[1] January CT, Wann LS, Calkins H, Chen LY, Cigarroa JE, Cleveland JC, Ellinor PT, Ezekowitz MD, Field ME, Furie KL, Heidenreich PA, Murray KT, Shea JB, Tracy CM, Yancy CW (2019). 2019 AHA/ACC/HRS focused update of the 2014 AHA/ACC/HRS guideline for the Management of Patients with Atrial Fibrillation: a report of the American College of Cardiology/American Heart Association task force on clinical practice guidelines and the Heart Rhythm Society. J Am Coll Cardiol.

[2] Goyal M, Demchuk AM, Menon BK, Eesa M, Rempel JL, Thornton J, Roy D, Jovin TG, Willinsky RA, Sapkota BL, Dowlatshahi D, Frei DF, Kamal NR, Montanera WJ, Poppe AY, Ryckborst KJ, Silver FL, Shuaib A, Tampieri D, Williams D, Bang OY, Baxter BW, Burns PA, Choe H, Heo JH, Holmstedt CA, Jankowitz B, Kelly M, Linares G, Mandzia JL, Shankar J, Sohn SI, Swartz RH, Barber PA, Coutts SB, Smith EE, Morrish WF, Weill A, Subramaniam S, Mitha AP, Wong JH, Lowerison MW, Sajobi TT, Hill MD (2015). Randomized assessment of rapid endovascular treatment of ischemic stroke. N Engl J Med.

[3] Campbell BC, Mitchell PJ, Kleinig TJ, Dewey HM, Churilov L, Yassi N, Yan B, Dowling RJ, Parsons MW, Oxley TJ, Wu TY, Brooks M, Simpson MA, Miteff F, Levi CR, Krause M, Harrington TJ, Faulder KC, Steinfort BS, Priglinger M, Ang T, Scroop R, Barber PA, McGuinness B, Wijeratne T, Phan TG, Chong W, Chandra RV, Bladin CF, Badve M, Rice H, de Villiers L, Ma H, Desmond PM, Donnan GA, Davis SM (2015). Endovascular therapy for ischemic stroke with perfusion-imaging selection. N Engl J Med.

[4] Berkhemer OA, Fransen PS, Beumer D, van den Berg LA, Lingsma HF, Yoo AJ, Schonewille WJ, Vos JA, Nederkoorn PJ, Wermer MJ, van Walderveen MA, Staals J, Hofmeijer J, van Oostayen JA, Nijeholt GJ L à, Boiten J, Brouwer PA, Emmer BJ, de Bruijn SF, van Dijk LC, Kappelle LJ, Lo RH, van Dijk EJ, de Vries J, de Kort PL, van Rooij WJ, van den Berg JS, van Hasselt BA, Aerden LA, Dallinga RJ, Visser MC, Bot JC, Vroomen PC, Eshghi O, Schreuder TH, Heijboer RJ, Keizer K, Tielbeek AV, den Hertog HM, Gerrits DG, van den Berg-Vos RM, Karas GB, Steyerberg EW, Flach HZ, Marquering HA, Sprengers ME, Jenniskens SF, Beenen LF, van den Berg R, Koudstaal PJ, van Zwam WH, Roos YB, van der Lugt A, van Oostenbrugge RJ, Majoie CB, Dippel DW (2015). A randomized trial of intraarterial treatment for acute ischemic stroke. N Engl J Med.

[5] Jovin TG, Chamorro A, Cobo E, de Miquel MA, Molina CA, Rovira A, San Román L, Serena J, Abilleira S, Ribó M, Millán M, Urra X, Cardona P, López-Cancio E, Tomasello A, Castaño C, Blasco J, Aja L, Dorado L, Quesada H, Rubiera M, Hernandez-Pérez M, Goyal M, Demchuk AM, von Kummer R, Gallofré M, Dávalos A (2015). Thrombectomy within 8 hours after symptom onset in ischemic stroke. N Engl J Med.

[6] Saver JL, Goyal M, Bonafe A, Diener HC, Levy EI, Pereira VM, Albers GW, Cognard C, Cohen DJ, Hacke W, Jansen O, Jovin TG, Mattle HP, Nogueira RG, Siddiqui AH, Yavagal DR, Baxter BW, Devlin TG, Lopes DK, Reddy VK, du Mesnil de Rochemont R, Singer OC, Jahan R (2015). Stent-retriever thrombectomy after intravenous t-PA vs. t-PA alone in stroke. N Engl J Med.

[7] Nogueira RG, Jadhav AP, Haussen DC, Bonafe A, Budzik RF, Bhuva P, Yavagal DR, Ribo M, Cognard C, Hanel RA, Sila CA, Hassan AE, Millan M, Levy EI, Mitchell P, Chen M, English JD, Shah QA, Silver FL, Pereira VM, Mehta BP, Baxter BW, Abraham MG, Cardona P, Veznedaroglu E, Hellinger FR, Feng L, Kirmani JF, Lopes DK, Jankowitz BT, Frankel MR, Costalat V, Vora NA, Yoo AJ, Malik AM, Furlan AJ, Rubiera M, Aghaebrahim A, Olivot JM, Tekle WG, Shields R, Graves T, Lewis RJ, Smith WS, Liebeskind DS, Saver JL, Jovin TG (2018). Thrombectomy 6 to 24 Hours after Stroke with a Mismatch between Deficit and Infarct. N Engl J Med.

[8] Albers GW, Marks MP, Kemp S, Christensen S, Tsai JP, Ortega-Gutierrez S, McTaggart RA, Torbey MT, Kim-Tenser M, Leslie-Mazwi T, Sarraj A, Kasner SE, Ansari SA, Yeatts SD, Hamilton S, Mlynash M, Heit JJ, Zaharchuk G, Kim S, Carrozzella J, Palesch YY, Demchuk AM, Bammer R, Lavori PW, Broderick JP, Lansberg MG (2018). Thrombectomy for stroke at 6 to 16 hours with selection by perfusion imaging. N Engl J Med.

[9] Lin S, Wu B, Hao ZL, Kong FY, Tao WD, Wang DR, He S, Liu M (2011). Characteristics, treatment and outcome of ischemic stroke with atrial fibrillation in a Chinese hospital-based stroke study. Cerebrovasc Dis.

[10] Kimura K, Minematsu K, Yamaguchi T (2005). Atrial fibrillation as a predictive factor for severe stroke and early death in 15,831 patients with acute ischaemic stroke. J Neurol Neurosurg Psychiatry.

[11] Henninger N, Goddeau RP, Karmarkar A, Helenius J, McManus DD (2016). Atrial fibrillation is associated with a worse 90-day outcome than other Cardioembolic stroke subtypes. Stroke..

[12] Steger C, Pratter A, Martinek-Bregel M, Avanzini M, Valentin A, Slany J, Stöllberger C (2004). Stroke patients with atrial fibrillation have a worse prognosis than patients without: data from the Austrian stroke registry. Eur Heart J.

[13] Yue R, Li D, Yu J, Li S, Ma Y, Huang S, Zeng Z, Zeng R, Sun X (2016). Atrial Fibrillation is Associated With Poor Outcomes in Thrombolyzed Patients With Acute Ischemic Stroke: A Systematic Review and Meta-Analysis. Medicine (Baltimore).

[14] Hu Y, Ji C (2021). Efficacy and safety of thrombolysis for acute ischemic stroke with atrial fibrillation: a meta-analysis. BMC Neurol.

[15] Powers WJ, Rabinstein AA, Ackerson T, Adeoye OM, Bambakidis NC, Becker K, Biller J, Brown M, Demaerschalk BM, Hoh B, Jauch EC, Kidwell CS, Leslie-Mazwi TM, Ovbiagele B, Scott PA, Sheth KN, Southerland AM, Summers DV, Tirschwell DL (2019). Guidelines for the Early Management of Patients With Acute Ischemic Stroke: 2019 update to the 2018 guidelines for the early Management of Acute Ischemic Stroke: a guideline for healthcare professionals from the American Heart Association/American Stroke Association. Stroke.

[16] Heshmatollah A, Fransen P, Berkhemer OA, Beumer D, van der Lugt A, Majoie C, Oostenbrugge RJ, van Zwam WH, Koudstaal PJ, Roos Y, Dippel D (2017). Endovascular thrombectomy in patients with acute ischaemic stroke and atrial fibrillation: a MR CLEAN subgroup analysis. EuroIntervention..

[17] Smaal JA, de Ridder IR, Heshmatollah A, van Zwam WH, Dippel D, Majoie CB, Brown S, Goyal M, Campbell B, Muir KW, Demchuck AM, Davalos A, Jovin TG, Mitchell PJ, White P, Saver JL, Hill MD, Roos YB, van der Lugt A, van Oostenbrugge RJ (2020). Effect of atrial fibrillation on endovascular thrombectomy for acute ischemic stroke. A meta-analysis of individual patient data from six randomised trials: results from the HERMES collaboration. Eur Stroke J.

[18] Huang K, Zha M, Gao J, Du J, Liu R, Liu X (2021). Increased intracranial hemorrhage of mechanical thrombectomy in acute ischemic stroke patients with atrial fibrillation. J Thromb Thrombolysis.

[19] Munir MB, Alqahtani F, Beltagy A, Tarabishy A, Alkhouli M (2017). Comparative outcomes of mechanical Thrombectomy for acute ischemic stroke in patients with and without atrial fibrillation. J Vasc Interv Radiol.

[20] Akbik F, Alawieh A, Cawley CM, Howard BM, Tong FC, Nahab F, et al. Differential effect of mechanical thrombectomy and intravenous thrombolysis in atrial fibrillation associated stroke. J Neurointerv Surg. 2021;13(10):883–8. 10.1136/neurintsurg-2020-016720. 10.1136/neurintsurg-2020-016720 PMC8377613 33318066

[21] Lin CJ, Luo CB, Chien C, Chang FC, Lin CJ, Lee IH, Hsu LC, Chung CP, Liu HY, Chi NF, How CK, Wang SJ, Guo WY, Lin YY (2020). Better endovascular mechanical thrombectomy outcome in atrial fibrillation patients with acute ischemic stroke: a single-center experience. J Chin Med Assoc.

[22] Jia B, Ren Z, Mokin M, Burgin WS, Bauer CT, Fiehler J, Mo D, Ma N, Gao F, Huo X, Luo G, Wang A, Pan Y, Song L, Sun X, Zhang X, Gui L, Song C, Peng Y, Wu J, Zhao S, Zhao J, Zhou Z, Li Y, Jing P, Yang L, Liu Y, Zhao Q, Liu Y, Peng X, Gao Q, Guo Z, Chen W, Li W, Cheng X, Xu Y, Zhang Y, Zhang G, Lu Y, Lu X, Wang D, Wang Y, Li H, Ling L, Peng G, Zhang J, Zhang K, Li S, Qi Z, Xu H, Tong X, Ma G, Liu R, Guo X, Deng Y, Leng X, Leung TW, Liebeskind DS, Wang Y, Wang Y, Miao Z (2021). Current Status of Endovascular Treatment for Acute Large Vessel Occlusion in China: A Real-World Nationwide Registry. Stroke.

[23] Zaidat OO, Yoo AJ, Khatri P, Tomsick TA, von Kummer R, Saver JL, Marks MP, Prabhakaran S, Kallmes DF, Fitzsimmons BF, Mocco J, Wardlaw JM, Barnwell SL, Jovin TG, Linfante I, Siddiqui AH, Alexander MJ, Hirsch JA, Wintermark M, Albers G, Woo HH, Heck DV, Lev M, Aviv R, Hacke W, Warach S, Broderick J, Derdeyn CP, Furlan A, Nogueira RG, Yavagal DR, Goyal M, Demchuk AM, Bendszus M, Liebeskind DS, Cerebral Angiographic Revascularization Grading (CARG) Collaborators, STIR Revascularization working group, STIR Thrombolysis in Cerebral Infarction (TICI) Task Force (2013). Recommendations on angiographic revascularization grading standards for acute ischemic stroke: a consensus statement. Stroke.

[24] von Kummer R, Broderick JP, Campbell BC (2015). The Heidelberg bleeding classification: classification of bleeding events after ischemic stroke and reperfusion therapy. Stroke.

[25] Tong X, Wang Y, Fiehler J, Bauer CT, Jia B, Zhang X, Huo X, Luo G, Wang A, Pan Y, Ma N, Gao F, Mo D, Song L, Sun X, Liu L, Deng Y, Li X, Wang B, Ma G, Wang Y, Ren Z, Miao Z (2021). Thrombectomy Versus Combined Thrombolysis and Thrombectomy in Patients With Acute Stroke: A Matched-Control Study. Stroke.

[26] Widimsky P (2017). Acute ischaemic stroke in atrial fibrillation: worse outcomes unrelated to treatment methods. EuroIntervention.

[27] Tan BY, Kong WY, Ngiam JN, Teoh HL, Sharma VK, Yeo LL (2017). The role of topographic collaterals in predicting functional outcome after thrombolysis in anterior circulation ischemic stroke. J Neuroimaging.

[28] Sun B, Shi Z, Pu J, Yang S, Wang H, Yang D, Hao Y, Lin M, Ke W, Liu W, Guo F, Bai Y, Zhang S, Li Z, Li S, Zuo M, Xu G, Zi W, Liu X (2019). Effects of mechanical thrombectomy for acute stroke patients with etiology of large artery atherosclerosis. J Neurol Sci.

[29] Guglielmi V, LeCouffe NE, Zinkstok SM, Compagne K, Eker R, Treurniet KM, Tolhuisen ML, van der Worp HB, Jansen I, van Oostenbrugge RJ, Marquering HA, Dippel D, Emmer BJ, Majoie C, Roos Y, Coutinho JM (2019). Collateral circulation and outcome in atherosclerotic versus Cardioembolic cerebral large vessel occlusion. Stroke.

[30] Wang Y, Zhao X, Liu L, Soo YO, Pu Y, Pan Y, Wang Y, Zou X, Leung TW, Cai Y, Bai Q, Wu Y, Wang C, Pan X, Luo B, Wong KS (2014). Prevalence and outcomes of symptomatic intracranial large artery stenoses and occlusions in China: the Chinese intracranial atherosclerosis (CICAS) study. Stroke.

[31] Yeo L, Bhogal P, Gopinathan A, Cunli Y, Tan B, Andersson T (2019). Why does mechanical Thrombectomy in large vessel occlusion sometimes fail? : A review of the literature. Clin Neuroradiol.

